# JUMPSTART pilot: assessing the acceptability and feasibility of a novel early mobilization program following transcatheter aortic valve replacement

**DOI:** 10.3389/fcvm.2025.1568844

**Published:** 2025-06-25

**Authors:** Marija Corovic, Karen Mosleh, Esha Karia, Sachi Chan, Olivia Puglisi, Jacob Crawshaw, Tasmiya Asif, Tej Sheth, James Velianou, Patrick Magloire, J. D. Schwalm, Madhu Natarajan

**Affiliations:** ^1^ Department of Medicine, McMaster University, Hamilton, ON, Canada; ^2^ Centre for Evidence-Based Implementation, Hamilton Health Sciences Knowledge Centre, Hamilton, ON, Canada; ^3^ Lynch School of Education and Human Development, Boston College, Chestnut Hill, MA, United States; ^4^ Nuffield Department of Primary Care Health Sciences, University of Oxford, Oxford, United Kingdom; ^5^ Centre for Implementation Research, Ottawa Hospital Research Institute, Ottawa, ON, Canada; ^6^ Department of Cardiology, Hamilton Health Sciences Corporation, Hamilton, ON, Canada; ^7^ Population Health Research Institute, Hamilton, ON, Canada

**Keywords:** aortic stenosis, aortic valve replacement, TAVR, TAVI, early mobilization, exercise program, virtual program

## Abstract

Patients undergoing a transcatheter aortic valve replacement (TAVR) are typically discharged from hospital the next day, leaving little time to support mobilization needs. The JUMPSTART program was developed as a self-directed, tailored and virtual exercise program to improve post-TAVR patients' mobilization. This pilot evaluation assessed the acceptability and feasibility of the preliminary exercise module developed for the program. The evaluation was conducted at a regional cardiac centre in Ontario, Canada. Patients meeting inclusion criteria were contacted via telephone post-discharge and provided with an electronic link to the JUMPSTART exercise module. A second call was made, 14 days post-discharge, to gather feedback regarding module acceptability and feasibility, and to discuss barriers to participation, via a structured survey. Out of the 165 eligible patients who answered the phone when called post-discharge, 112 (68%) completed the survey. A major barrier to participating in the survey evaluation was the technological requirement. Sixty-eight respondents (61%) had done the recommended exercises; they were satisfied with the module (mean = 5.92; 1 = very dissatisfied and 7 = very satisfied) and most rated the exercises as being the “right level of difficulty” (56%). For the 44/112 (39%) who did not try the exercises, key barriers were being busy (*n* = 13), not feeling well (n = 10), and believing the module was unnecessary (*n* = 8). The preliminary JUMPSTART module was determined to be acceptable and feasible by TAVR patients who attempted the exercises. Findings refined the implementation of the JUMPSTART program, which has been expanded to include additional modules and is undergoing a comprehensive program evaluation.

## 1 Introduction

Transcatheter aortic valve replacement (TAVR), also referred to as transcatheter aortic valve intervention (TAVI) is currently considered the leading treatment option for most individuals with severe aortic stenosis (1–3). Differences in post-TAVR discharge practices exist around the world; the standard care pathway in Southern Ontario, Canada, enables same-day ambulation and allows next-day discharge (i.e., an overnight model) with excellent safety and efficacy outcomes (4–6). TAVR patients typically range in age from 65 to 95 years of age. Most experience other health conditions, in addition to aortic stenosis, including risk factors for future cardiac events such as hypertension, diabetes, and dyslipidemia, as well as geriatric syndromes, which are associated with adverse outcomes such as falling, frailty and cognitive and physical decline (7).

Cardiac rehabilitation is a well-recognized resource for cardiovascular patients. TAVR patients benefit from cardiac rehabilitation despite being of an older age and experiencing comorbidities (8–10). However, a recent review, which included over 3,300 TAVR patients from 24 hospitals, stated that only 30.6% had attended cardiac rehabilitation by 90 days post-discharge (11). Surgical patients' and general cardiac patients' participation rates have been reported to be much higher, at 43% and 57%, respectively (12, 13). Furthermore, the Million Hearts Cardiac Rehabilitation Collaborative promotes a goal of 70% attendance for cardiac rehabilitation, for all cardiac patients (14). Cardiac rehabilitation programs are noticeably being underutilized by TAVR patients (15). The COVID-19 pandemic further restricted the accessibility and attendance of conventional cardiac rehabilitation programs (16). Thus, clinical and research interests have shifted to understanding and optimizing virtual approaches to cardiac rehabilitation (17).

A regional cardiac centre in Southern Ontario, Canada, has one of the largest TAVR clinics in Canada, performing over 350 procedures per year. In November 2019, the overnight model was implemented at the cardiac centre and has since become standard care practice (4, 18). Approximately 80% of this centre's patients are ambulatory on the same day as their procedure and are discharged the following day. Early mobilization, to facilitate patients' return to their baseline activity levels, is imperative for a safe, accelerated discharge, and for positive health outcomes (18). Furthermore, it is known that sedentary patients experience a greater risk of functional decline and mortality, one-year post-TAVR procedure (19); however, there is no existing standard early mobilization protocol or exercise therapy available for patients. Many TAVR patients at the centre and their family members sought advice from hospital staff regarding recommendations for safe and appropriate physical activity post-TAVR. An informal survey was conducted with TAVR patients by clinical fellows at 30 days post-discharge (March to May 2021); nearly all of the approximately 35 patients surveyed expressed an interest in participating in a structured home-based exercise program, if offered.

Therefore, the JUMPSTART program was created to support post-TAVR patients' early mobilization and their return to baseline physical activity levels by offering structured and safe low-impact exercise modules. The program is self-directed, tailored to the patient population, and virtual/home-based. The virtual format aligns with public preferences and health service limitations initially implemented during the COVID-19 pandemic. The program is not a substitute for cardiac rehabilitation, but rather a precursor or complement. All suitable TAVR patients are encouraged to participate in both the JUMPSTART program as well as a cardiac rehabilitation program. The objective of this paper is to describe a pilot evaluation which assessed the acceptability and feasibility of the JUMPSTART program's preliminary exercise module, including any barriers to engagement in the program.

## 2 Methods

### 2.1 Evaluation design

This was a prospective, observational, and non-randomized pilot evaluation. Ethics approval was waived by the Hamilton Integrated Research Ethics Board, as the JUMPSTART program was offered to all eligible patients as a quality initiative.

### 2.2 Setting and participants

The evaluation was conducted at a regional cardiac centre located in Southern Ontario, Canada. Participants were included if they were outpatients who had undergone a TAVR procedure, were managed through the TAVR overnight model, successfully completed an ambulation assessment prior to discharge (i.e., modified Timed Up and Go and 2 Minute Walk tests) and were deemed eligible for next-day discharge post-procedure. Patients were excluded if they met any of the following criteria: were inpatients (i.e., hospitalized after the procedure); had a non-transfemoral approach for TAVR; had a pacemaker temporarily left in at the end of their procedure; or received a permanent pacemaker within one month before their procedure. 

### 2.3 Intervention

The JUMPSTART program consists of virtual modules (i.e., instructional exercise videos) of varying levels of intensity, which are approximately 20 minutes long. Each module is comprised of a brief introduction, a warm-up, a series of low intensity strength, balance and coordination exercises, and a cool-down. Modules were developed by the evaluation team, in consultation with a cardiac rehabilitation physiotherapist and a cardiac rehabilitation specialist within the cardiac services of the hospital. They were developed to be safe and appropriate for the TAVR patient population; however, the JUMPSTART exercises are not exclusive to TAVR patients. This paper reports on the acceptability and feasibility of the preliminary exercise module that was created (exercises shown in Supplementary File 1). All patients were provided with a Patient Information Letter prior to discharge.

### 2.4 Survey recruitment

Patients were contacted by telephone by a hospital administrative assistant five days after being discharged from the hospital. The purpose of the call was to review patients' clinical recovery, and to introduce them to the JUMPSTART pilot if they met the eligibility criteria. If they agreed to participate in the evaluation survey, they were provided with an electronic link to the exercise module and were encouraged to try it at home; all patients received the same instructions. A second telephone call was made to participants 14 days after their discharge, by the same administrative assistant, to administer the brief survey.

### 2.5 Survey development

The survey was developed by the evaluation team (Supplementary File 2); a formal framework was not used. The survey consisted of five multiple choice questions, three open-ended response questions, and one Likert-scale response question (1 = very dissatisfied, 7 = very satisfied). Questions addressed module uptake, participant experience and satisfaction, and recommendations for improvement. Participation tracking and survey responses were saved on a secure shared hospital drive.

### 2.6 Data analysis

Data were analyzed by MC and reviewed by KM, MN, and JC. Quantitative data were analyzed using Microsoft Excel. For patients' clinical characteristics, z-tests were used for proportion comparisons and independent t-tests were used for mean age comparisons. Multiple-choice survey question analysis involved number counts and percentages/proportions. The Likert scale responses were presented as a mean ± standard deviation (SD), and median. Qualitative survey data were available for two of the three open-ended questions. Responses were concise and direct; therefore, they did not require extensive thematic analysis.

## 3 Results

### 3.1 Survey data collection

Data were collected between January 6, 2022, to March 2, 2023. During that period, 265 patients were managed through the TAVR overnight model and 216 met eligibility criteria. Of those that were eligible, 165 answered the clinical follow-up call and were offered the program (i.e., provided with an electronic link to the exercise module). Ultimately, 112/165 (68%) agreed to participate in the evaluation and completed the survey. The primary reasons for not participating in the evaluation survey were lack of experience with computers or not having a computer (*n* = 25; 52%) and already exercising independently and therefore not requiring instruction (*n* = 17; 35%). Recruitment and participation are summarized in Supplementary File 3.

### 3.2 Baseline clinical characteristics

Table 1 compares the characteristics of survey respondents (i.e., JUMPSTART-eligible patients who completed the survey; *n* = 112) with non-participants (i.e., eligible patients who chose not to participate, *n* = 48; or who did not answer telephone calls, *n* = 56). A significantly higher proportion of survey respondents were smokers compared with people who chose not to participate; while a significantly higher proportion of people who did not answer telephone calls had diabetes compared to survey respondents, and a significantly higher proportion of people who chose not to participate had dyslipidemia compared with survey respondents. Table 2 compares the characteristics of survey respondents who attempted the JUMPSTART exercises (*n* = 68) with participants who did not try the exercises (*n* = 44). There were no significant differences between these two groups. The mean age of the survey respondents was 80 years, and 47% were female.

**Table 1:** Eligible patients' clinical characteristics (*n* = 216): comparison of those who completed the survey evaluation with those who did not.

Clinical characteristics	Survey respondents (*n* = 112)	Non-respondents (did not answer phone) (*n* = 56)	*P*-value^a^	Non-respondents (declined to participate) (*n* = 48)	*P*-value^a^	Combined/Total (*n* = 216)
Mean age (years)	80.2 (SD = 7.0)	79.9 (SD = 6.7)	0.69	80.1 (SD = 5.9)	0.90	80.1 (SD = 6.6)
Female	53 (47%)	26 (46%)	0.91	21 (44%)	0.67	100 (46%)
New York Heart Association Classification						
1	25 (22%)	11 (20%)	0.69	9 (19%)	0.61	45 (21%)
2	67 (60%)	30 (54%)	0.44	26 (54%)	0.51	123 (57%)
≥3	20 (18%)	15 (27%)		13 (27%)		48 (22%)
Diabetes	29 (26%)	25 (45%)	0.01*	19 (40%)	0.08	73 (34%)
Hypertension	92 (82%)	46 (82%)	1.00	45 (94%)	0.05	183 (85%)
Dyslipidemia	83 (74%)	42 (75%)	0.90	44 (92%)	0.01*	169 (78%)
Smoking	38 (34%)	11 (20%)	0.05	8 (17%)	0.03*	57 (26%)
CAD	26 (23%)	13 (23%)	1.00	12 (25%)	0.81	51 (24%)
Atrial Fibrillation	42 (38%)	14 (25%)	0.11	15 (31%)	0.45	71 (33%)
Stroke	8 (7%)	4 (7%)	1.00	2 (4%)	0.48	14 (6%)
PAD	10 (9%)	1 (2%)	0.08	2 (4%)	0.29	13 (6%)
COPD	15 (13%)	6 (12%)	0.62	4 (8%)	0.36	25 (12%)

^a^ Compared to survey respondents.

* Statistically significant (*p* < 0.05).

**Table 2:** Survey respondents' clinical characteristics (*n* = 112): comparison of those who attempted the JUMPSTART exercise module with those who did not.

Clinical characteristics	Attempted module (*n* = 68)	Did not attempt (*n* = 44)	*P*-value
Mean age (years)	80.0 (SD = 6.6)	80.6 (SD = 6.6)	0.64
Female	35 (51%)	18 (41%)	0.28
New York Heart Association Classification			
1	13 (19%)	12 (27%)	0.31
2	41 (60%)	26 (59%)	0.90
≥3	14 (21%)	6 (14%)	0.35
Diabetes	16 (23%)	13 (29%)	0.48
Hypertension	58 (85%)	34 (77%)	0.28
Lipids	51 (75%)	32 (73%)	0.79
Smoking	25 (37%)	13 (29%)	0.43
Any CAD	18 (26%)	8 (18%)	0.31
Atrial Fibrillation	23 (34%)	19 (43%)	0.32
Stroke	3 (4%)	5 (11%)	0.16
PAD	5 (7%)	5 (11%)	0.46
COPD	9 (13%)	6 (14%)	0.95

### 3.3 Survey responses

Participants were asked if they watched the video and if they did the exercise program. Eighty-seven (78%) survey respondents reported watching the video and 68 (61%) stated that they completed the exercises. For the 44 (39%) that did not do the exercises, 13 specified that they were too busy but are interested in trying it in the future, 10 were feeling unwell and unable to exercise, eight believed that they are active enough on their own and do not benefit from an instructional exercise video, six did not receive the electronic link (i.e., email was likely delivered to a junk mail folder), four experienced technical difficulties, two felt intimidated to attempt the exercises, and one person reported that their Holter monitor made them feel uncomfortable.

Several questions were targeted at participants who completed the exercise module (*n* = 68). These individuals reported being satisfied with the module (mean = 5.9 (SD = 1.2); median = 6; 1 = very dissatisfied and 7 = very satisfied). Sixty-one respondents provided an answer when asked about the level of difficulty of the exercises in the module; 34 (56%) stated that the exercises were the “right level of difficulty”, while 21 (34%) thought they were “too easy” and six (10%) said they were “too difficult”. Only two people reported a physical limitation when doing JUMPSTART exercises; one was shortness of breath, and the other was discomfort in the groin area. When asked about specific recommendations for improving the module, eight comments were made; five respondents stated that the module was too easy, one suggested including guidance on when to progress to a higher intensity video, and two comments were specific to video voiceover instructions. When asked if they would recommend the JUMPSTART exercise program to others who have had a TAVR procedure, all of those who answered the question (*n* = 60; 100%) said yes. Eight responses were not reported for this final survey question; therefore, the denominator was 60 rather than 68.

## 4 Discussion

The JUMPSTART program is a structured, home-based program, created to support early mobilization for post-TAVR patients. It is not a cardiac rehabilitation program, but it does focus on the same patient population, with similar goals. A recently published study assessed the feasibility of exercise-based cardiac telerehabilitation programs for post-TAVR patients (20, 21). It was reported that web-based telerehabilitation may not be feasible, primarily due to technical issues that were encountered; however, it was also reported that telerehabilitation can benefit patients by empowering them to be independent and supporting their adherence to physical training (20, 21). The JUMPSTART pilot evaluation assessed the acceptability and feasibility of the program's preliminary exercise module. Findings similarly concluded that use of technology is a fundamental barrier to participation and must be mitigated. Nonetheless, over half of the patients who were eligible for the JUMPSTART program (61%) attempted the exercise module. Participants were satisfied with the module, they agreed that the program would be beneficial to post-TAVR patients, and they did not raise any major safety concerns. In systems of care where patients are routinely admitted post-TAVR for more than one day, there might be opportunities to initiate early mobilization education in-hospital, coordinated by physiotherapists and then directed to a self-directed program such as JUMPSTART after discharge.

### 4.1 Barriers to participation and subsequent program refinement

During the evaluation, there were eligible patients who declined to participate in the evaluation survey, along with survey participants who reported that they did not do the exercises. The rationale given by these two groups provided insight into various barriers to participating in a virtual program post TAVR.

Lack of experience with a computer or not owning a computer was a common justification for not participating in the evaluation survey. The TAVR patient population is almost exclusively older adults, with a mean age of over 81 years (22). Current research reports that only 61% of older adults own a smartphone and less than 50% own a tablet computer (23). Thus, limited access/use of technology was an expected barrier. This, however, has been mitigated by offering exercise modules through a conventional means (i.e., paper, as in Supplementary File 1). Instructional handouts, which include both text and images displaying the exercises, are now available to patients. Introducing the opportunity to participate in the JUMPSTART program and distributing handouts during pre-admission could increase patient participation. In addition, many TAVR patients spend time recovering at the home of a relative, therefore, there may be future opportunities to engage caregivers and family members in supporting and encouraging patient participation in the program.

Some respondents that reported being self-guided in terms of their physical activity and/or having an established exercise regiment, also chose not to take part in the program. JUMPSTART is intended to encourage early mobilization and provide advice on safe exercise. Therefore, although this issue is a barrier to participating in the preliminary JUMPSTART module, it is not a barrier to early mobilization. Since the completion of the pilot evaluation, the program has been expanded to include three additional exercise modules, with increasing levels of difficulty, to promote participation.

Some patients reported not feeling well enough to engage in JUMPSTART exercises. The TAVR patient population experiences complex health issues and comorbidities. Patients may have to prioritize other health-related issues, aside from engaging in physical activity, shortly after their TAVR procedure. In addition, a few respondents indicated that lack of time was a barrier; however, they expressed an intention to participate in the future. The evaluation follow-up call was made 14 days post-TAVR, and it is reasonable for patients to have had other priorities during that period.

Less common problems such as not receiving the email which contained the electronic link, and experiencing technical difficulties, have since been addressed. The link for the exercise modules is now included on a TAVR Recovery Handout which is distributed to all patients, both at a pre-TAVR clinic and in the recovery unit after their procedure. Physical handouts of the exercise instructions can be requested. In addition, eligible patients are now encouraged to attend a weekly, virtual, group-based JUMPSTART exercise session which is led by a cardiac rehabilitation physiotherapist.

### 4.2 Next phase

The evaluation findings have led to the establishment of a more comprehensive JUMPSTART research study, which is currently in progress. The study protocol has been registered on clinicaltrials.gov (NCT06040398). This research study involves a comprehensive evaluation of the program's impact on quality of life using the Toronto Aortic Stenosis Quality of Life Scale (24), along with program adoption, acceptability, feasibility, and barriers to participation. In addition, clinically meaningful encounters with cardiac rehabilitation programs are being evaluated, as the JUMPSTART program has been combined with rapid access to a hybrid (i.e., virtual and in-person) cardiac rehabilitation program. Most of these measures are being assessed at two weeks, and three months, post-TAVR.

### 4.3 Strengths and limitations

The JUMPSTART program is virtual and self-directed, allowing patients to access modules and engage in exercising at their convenience. Therefore, scheduling conflicts, and cost of transportation and/or parking are not barriers to participation. In addition, physiotherapists are not required to complete additional work for the purposes of this program, such as creating individualized early mobilization plans, or leading in-person educational or instructional exercise sessions. Therefore, the major strengths of the JUMPSTART program are its simplicity and cost-effectiveness, for both patients and the hospital or cardiac centre. The program could certainly be implemented as an extension of post-TAVR care in almost any health care system. As was stated in the Discussion section, the virtual format can also be a limitation for some patients. To mitigate this issue, physical (i.e., paper) copies of the exercises are available as part of the current program.

Regarding the limitations of the evaluation, among the 51 eligible patients who did not answer the clinical follow-up call (Supplementary File 3), many were readmitted to hospital and/or experienced other medical issues. Gathering complete data on those individual cases may have helped to identify predictors for engaging in early mobilization. However, detailed information had not been collected. Volunteer bias may be another possible limitation, as there were notable trends in the findings (Table 1). Patients who were more motivated for rehabilitation or more familiar with technology might have been more likely to engage with the program. Additionally, without a control group, the pilot evaluation could not determine whether JUMPSTART participation directly leads to improved outcomes. Lastly, the pilot evaluation survey was brief, which limited the available data and, consequently, the findings. However, the ongoing JUMPSTART research study will provide more comprehensive data on the current program's adoption, satisfaction, effectiveness, and implementation.

## 5 Conclusion

Patients undergoing TAVR are typically discharged from hospital the day after their procedure, leaving little time to support their mobilization needs. The JUMPSTART program is a novel, virtual early mobilization program, consisting of exercise modules of varying intensity levels for the post-TAVR patient population. A key barrier to participating in this evaluation was the technological requirement. However, over 60% of evaluation participants attempted the preliminary exercise module developed for the program, and a high degree of acceptability and feasibility was demonstrated amongst this group. A barrier assessment led to local program improvements and informed the development of a larger, ongoing research study. The JUMPSTART program has the potential to improve patient outcomes by providing an accessible, tailored, early mobilization protocol post-TAVR.

## Data Availability

The raw data supporting the conclusions of this article will be made available by the authors, upon reasonable request.
