# Supplementary material for: JUMPSTART pilot: assessing the acceptability and feasibility of a novel early mobilization program following transcatheter aortic valve replacement
Source: Front Cardiovasc Med. 2025 Jun 25;12:1568844. doi: 10.3389/fcvm.2025.1568844 (PMC12237925; doi:10.3389/fcvm.2025.1568844)
Supplement: Supplementary file 1 [file Datasheet1.pdf]

Jump Start TAVR

Early Mobilization Post Trans-catheter Aortic Valve Replacement(TAVR) Procedure

Watching the Video

Laptop in spacious area, Pad/Tablet propped up, or TV/Monitor with decluttered area in front. Visit the link **hhscebi.ca/projects/jumpstart**

Warm Up

1) Seated Arm Circles

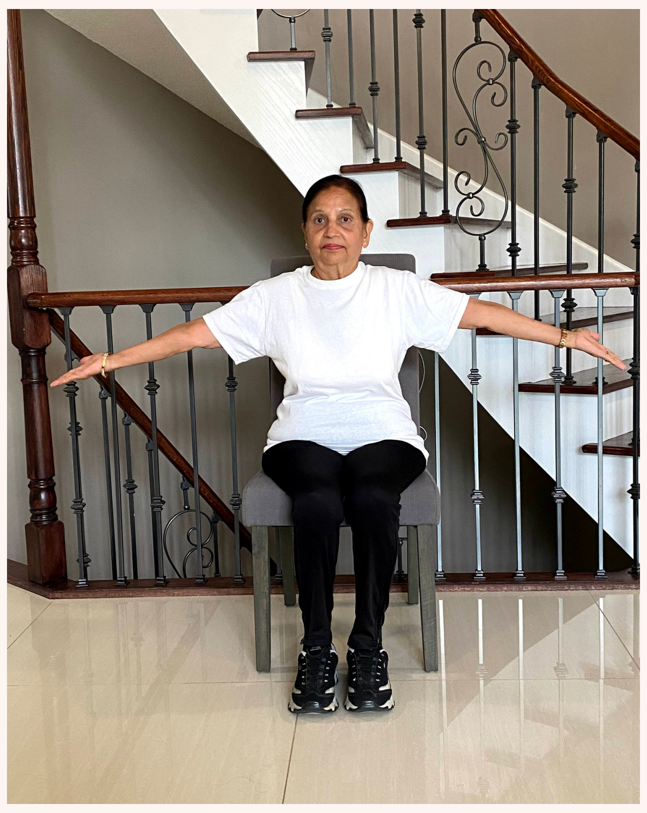

Extend your arms by your sides, rotate them 5 times forward & 5 times backwards

2) Seated Jacks

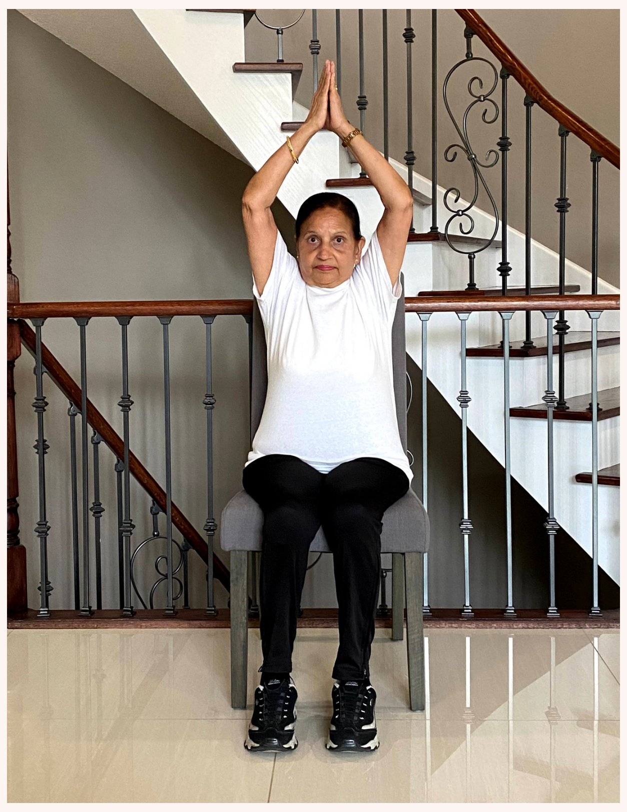

Extend your arms by your sides, clap your hands above your head 5 times

3) Seated Forward Curl

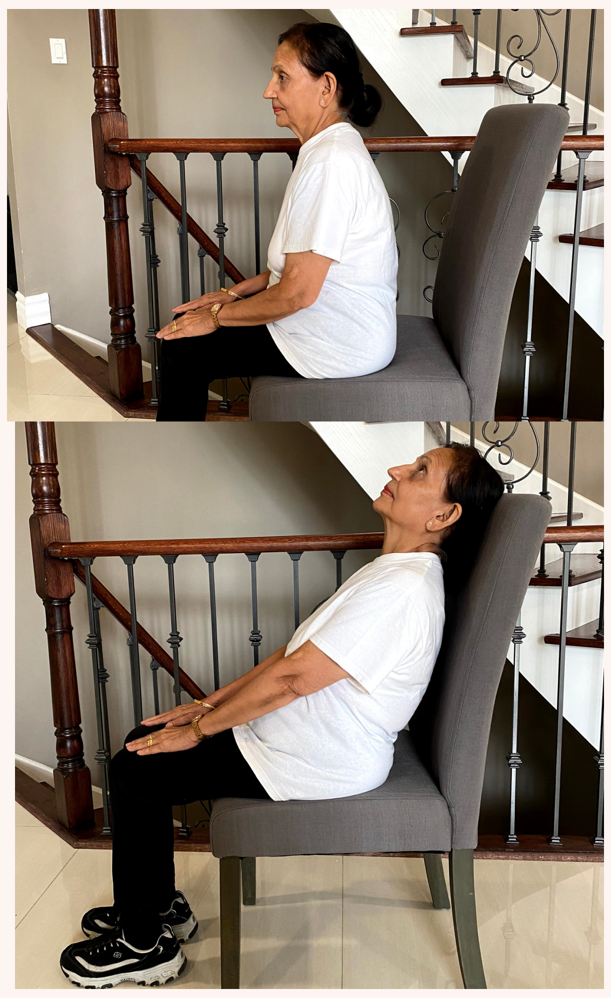

Sit in the middle of your chair, lean back until heels lift off the ground. Repeat 5 times

4) Seated Ankle Circles

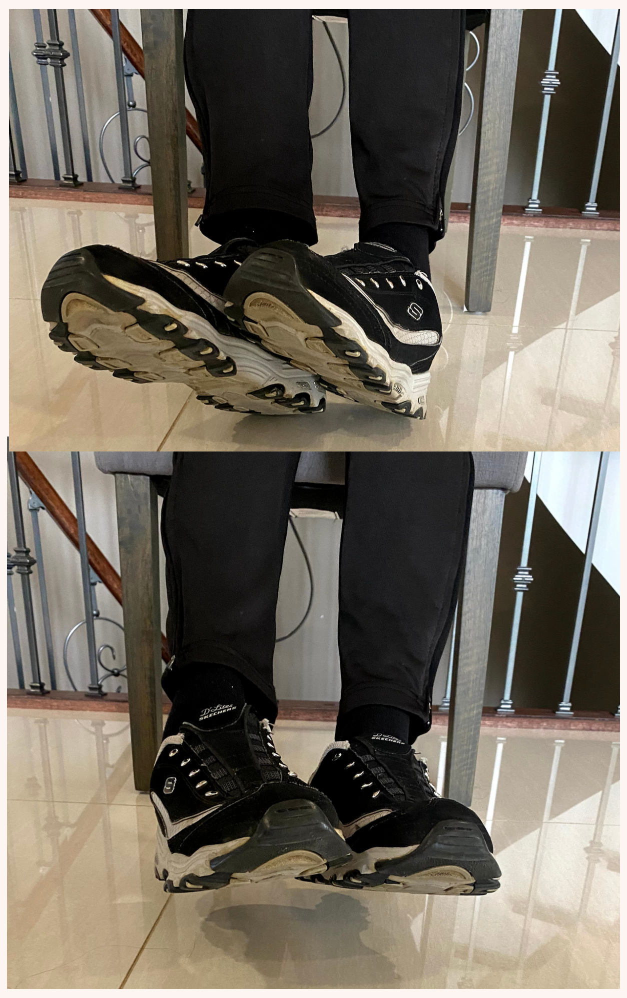

Rotate your ankles 5 times in one direction & 5 times in the opposite direction

Hamilton Health Sciences

This program is intended for a specific quality improvement project, and should NOT be shared or distributed to other people.

McMaster University

Exercises

1) Head Movements

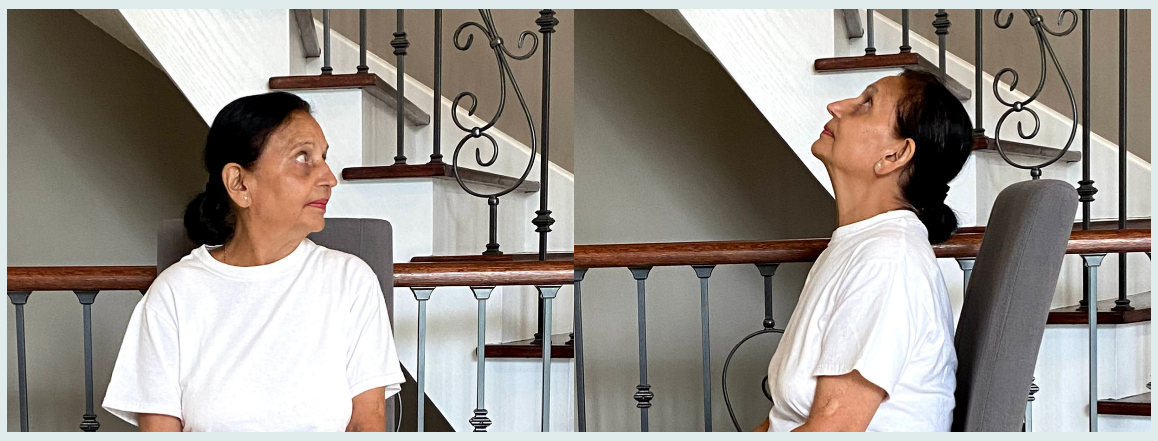

Tilt your head up & down 10 times & side to side 10 times

2) Shoulder Shrugs

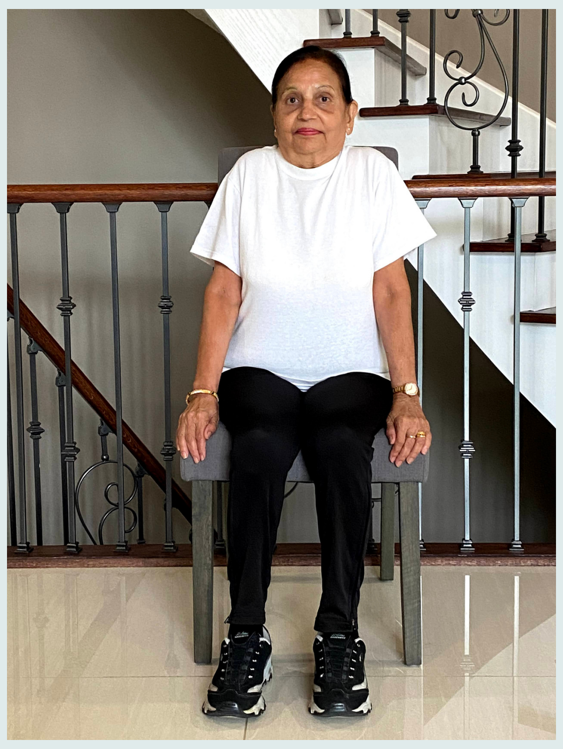

Lift your shoulders up & down 5 times

3) Vertical Seated Row

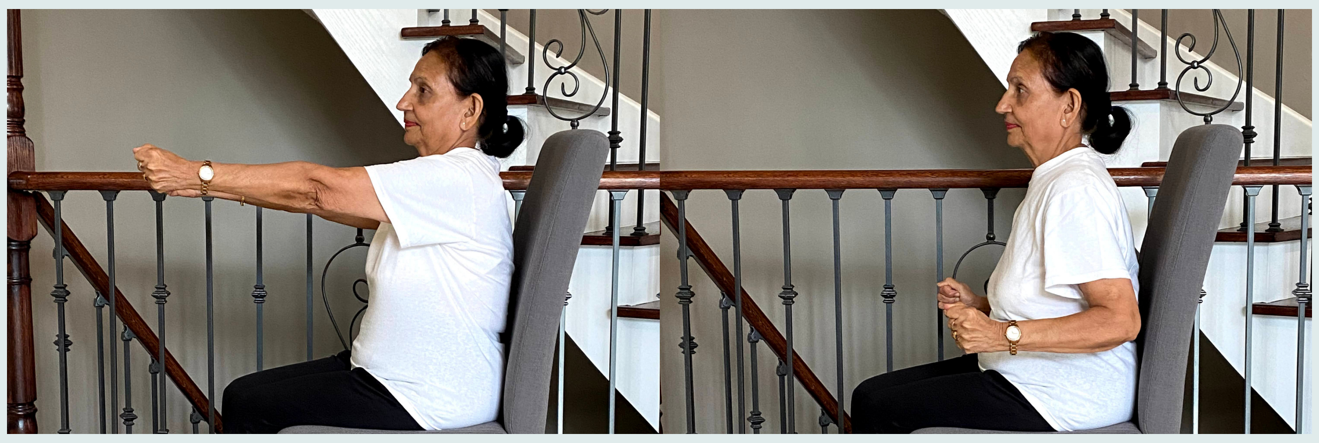

Extend your arms in front of you, bring them by your side. Repeat 5 times

4) Woodchops

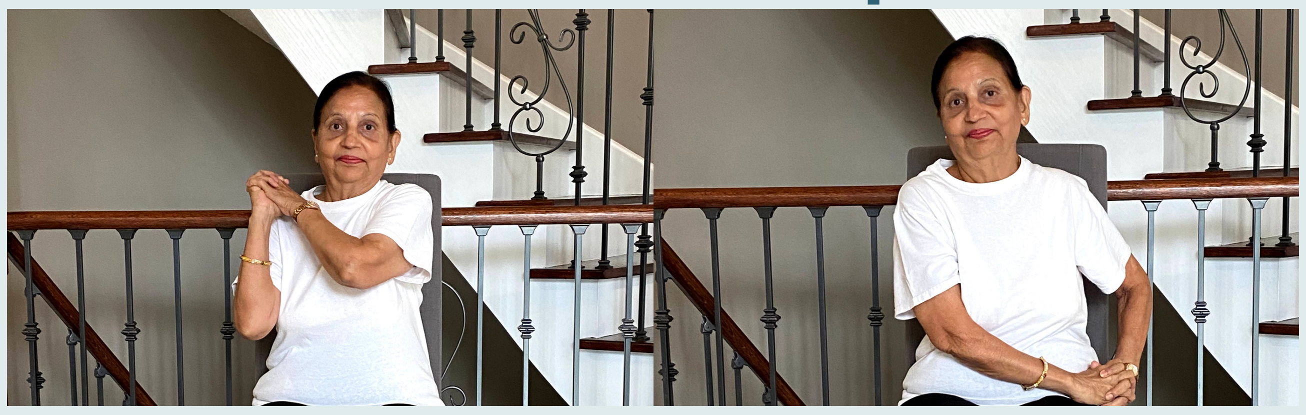

Put your hands together, move hands from shoulder to opposite side hip 5 times. Move to other shoulder & repeat 5 times

5) Seated Toe Taps

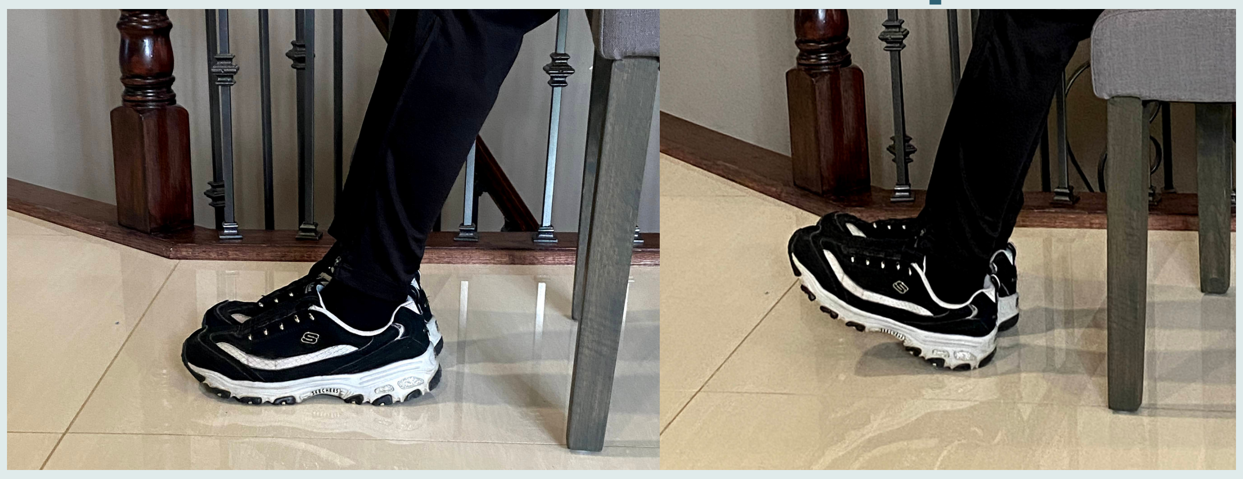

Sit far back in your chair, lift up toes as high as you can & bring them back down. Repeat 5 times

6) Half Squats with chair

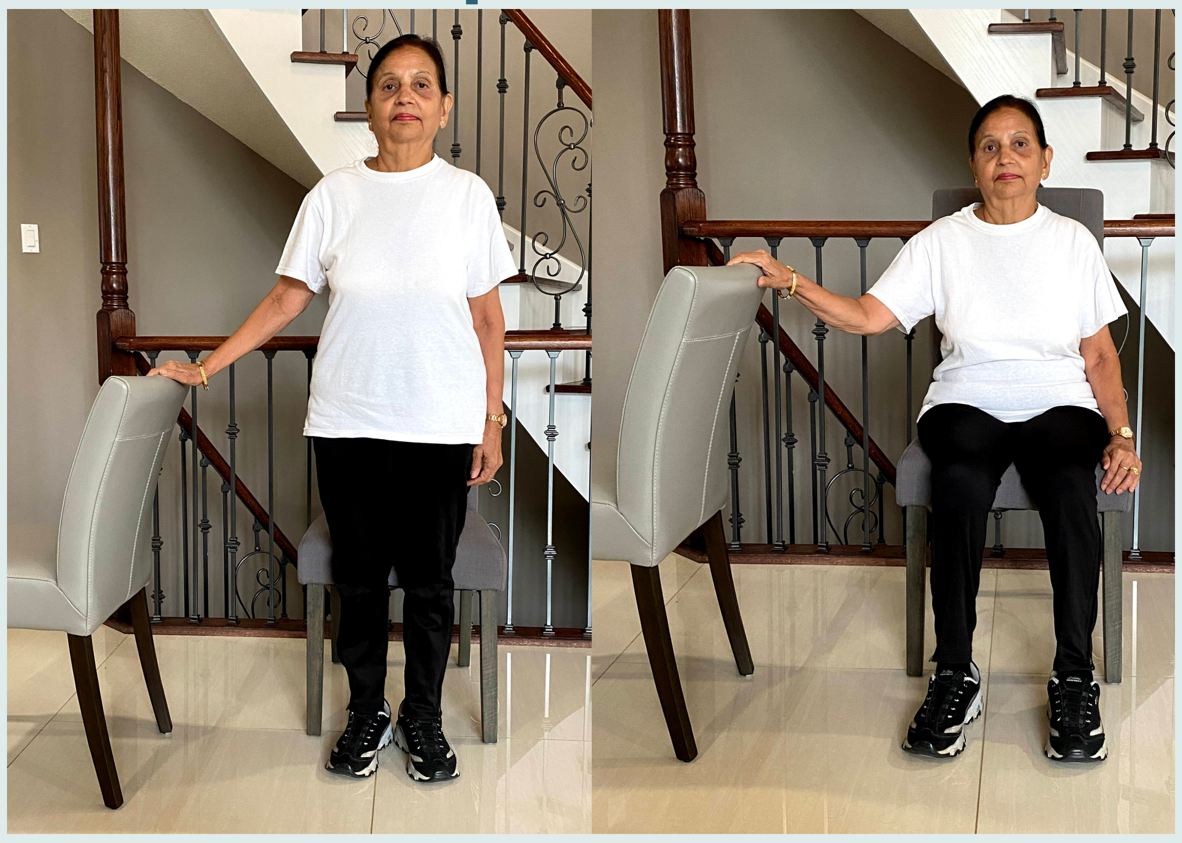

Put stable 4-legged chair against wall.

Stand in front of chair with feet shoulder-width apart.

Sit down in chair, then stand up again (hold onto another chair/wall/counter for support).

Repeat 5 times

7) Standing Marches with chair

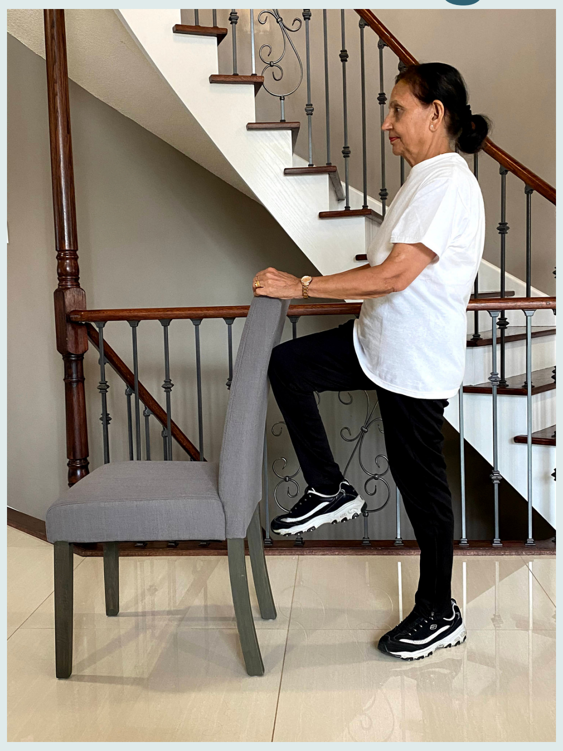

Place chair in front to hold for support. Raise knee & touch the back of chair, then bring leg back down. Alternate 5 times per leg

Hamilton Health Sciences

Any time you experience any discomfort, dizziness, shortness of breath, or chest pain, please sit down and stop the program

McMaster University

Cool Down

1) Shoulder Stretch

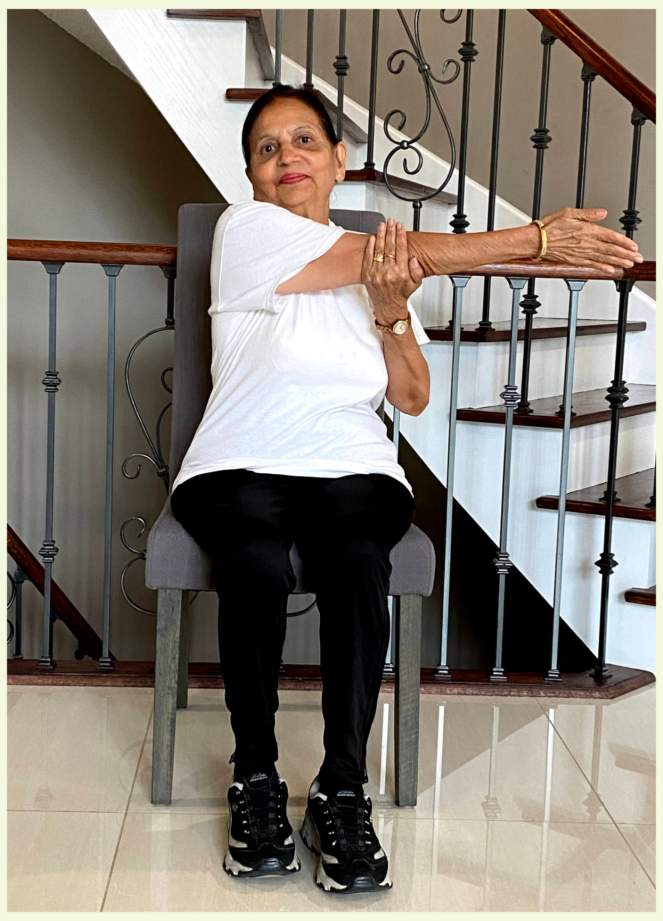

Bring your arm across your body, hold your shoulder or your elbow for 15 seconds. Switch to other arm & hold for 15 seconds

2) Knee to Chest Stretch

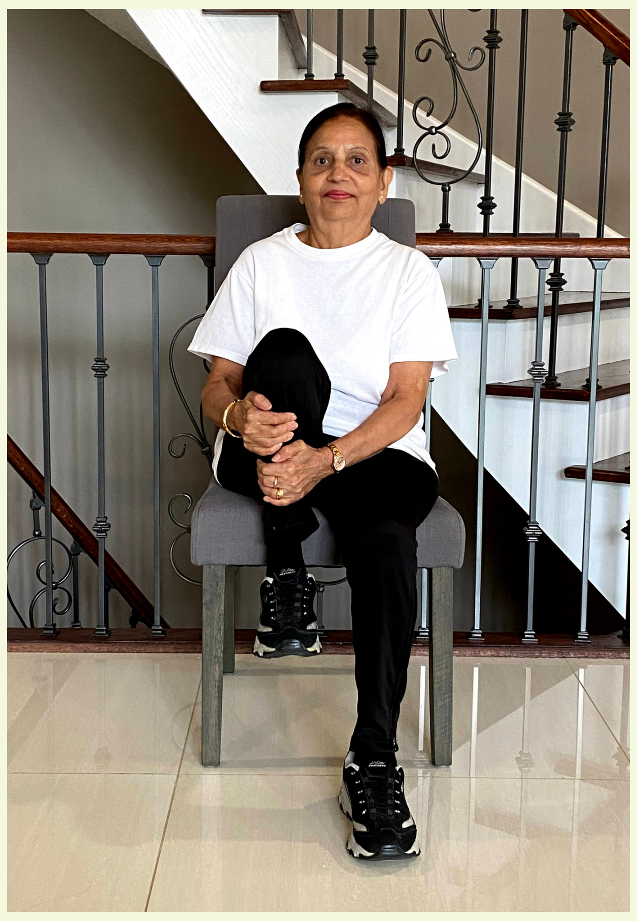

Lift up your knee towards your chest for 15 seconds. Switch legs & hold for 15 seconds.

3) Calf Stretch

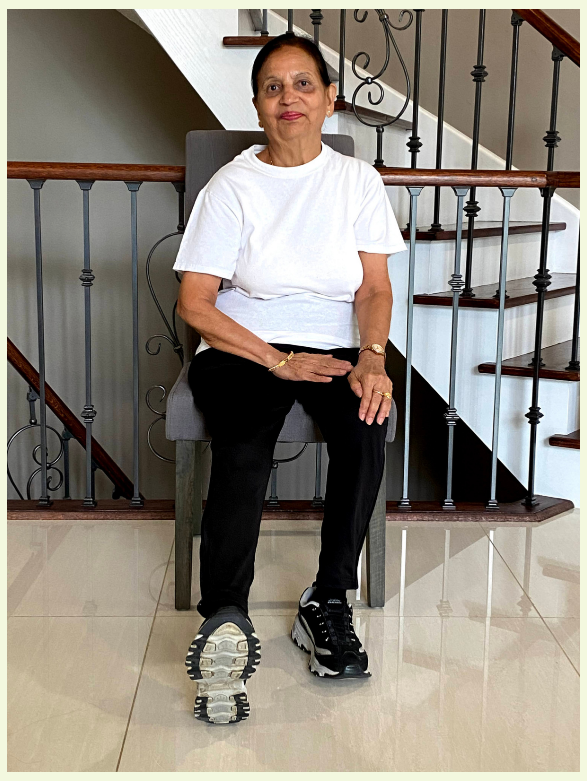

Sit in the middle of your chair, extend one leg pointing your toes upward & hold for 15 seconds. Switch legs & hold for 15 seconds.

4) Chest Stretch

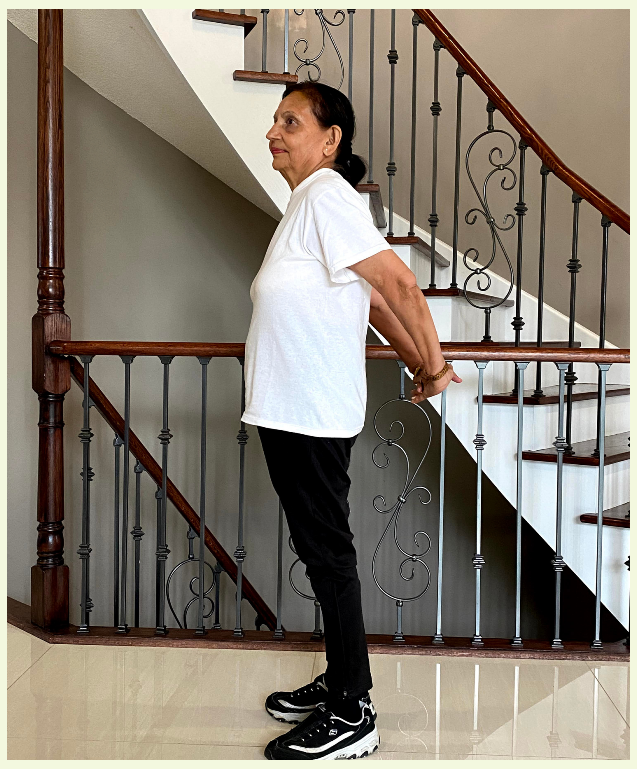

Stand up, bring your hands behind your back & try to touch them. Reach further back for a greater stretch. Hold for 15 seconds

5) Three Deep Breaths

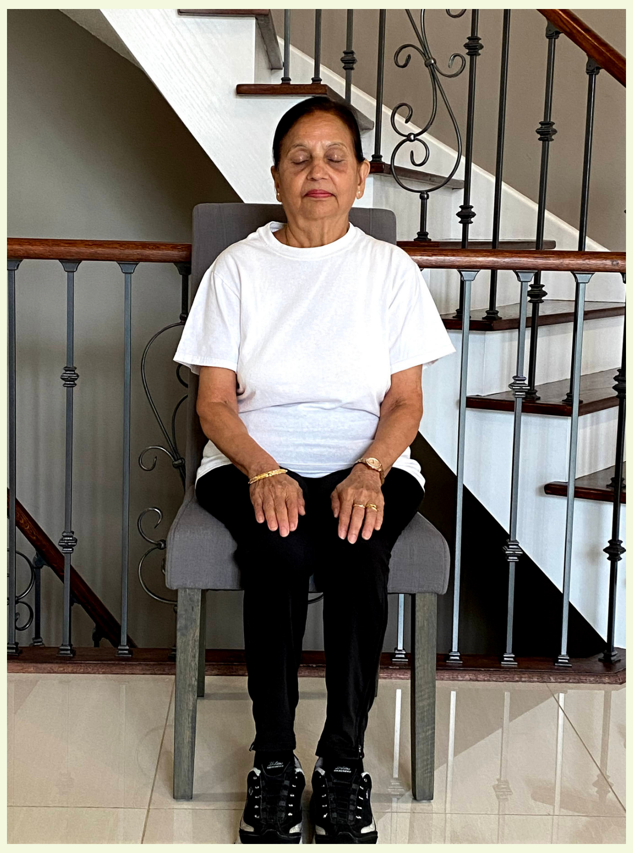

Close your eyes. Take a deep breath in expanding your belly, then breath out. Repeat 3 times

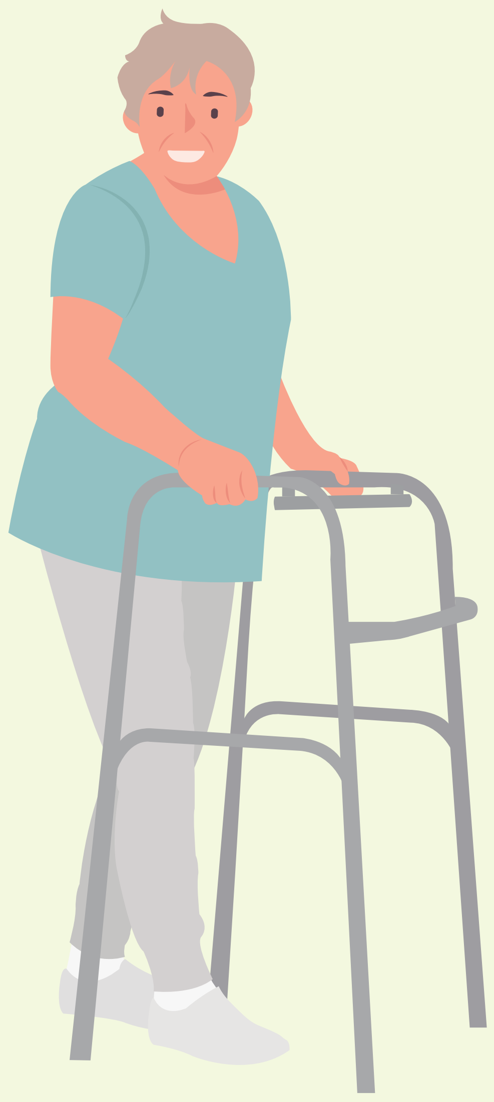

Follow the program with 10 minutes of walking inside or outside

Hamilton Health Sciences

McMaster University
