# Supplementary material for: JUMPSTART pilot: assessing the acceptability and feasibility of a novel early mobilization program following transcatheter aortic valve replacement
Source: Front Cardiovasc Med. 2025 Jun 25;12:1568844. doi: 10.3389/fcvm.2025.1568844 (PMC12237925; doi:10.3389/fcvm.2025.1568844)
Supplement: Supplementary file 2 [file Datasheet2.docx]

**Supplemental File 2:** JUMPSTART 14-day follow-up survey questions

1. Did you watch the JUMPSTART exercise program video?

- Yes
- No

2. Did you do the JUMPSTART exercise program?

- Yes
- No

3. *(If answer to Q2 is “No”)* Why not? (Open text response)

4. *(If answer to Q2 is “Yes”)* How would you rate your level of satisfaction with the JUMPSTART video on a scale of 1 to 7?

- 1 = Very dissatisfied
- 2 = Dissatisfied
- 3 = Somewhat dissatisfied
- 4 = Neither dissatisfied nor satisfied
- 5 = Somewhat satisfied
- 6 = Satisfied
- 7 = Very satisfied

5. Do you have any suggestions for improving the video? (Open text response)

6. Did you have any negative experiences while doing the JUMPSTART exercise program? Please select all that apply:

- I had chest pain
- I had shortness of breath
- I felt lightheaded
- I felt unsteady
- I injured myself
- Other (please describe)
- None

7. How would you rate the level of difficulty of the exercises that you did?

- Too difficult
- The right level of difficulty
- Too easy

8. Would you recommend the JUMPSTART exercise program to other people who have had TAVR?

- Yes
- No

9. *(If answer to Q8 is “No”)* Why not? (Open text response)
