# Supplementary material for: JUMPSTART pilot: assessing the acceptability and feasibility of a novel early mobilization program following transcatheter aortic valve replacement
Source: Front Cardiovasc Med. 2025 Jun 25;12:1568844. doi: 10.3389/fcvm.2025.1568844 (PMC12237925; doi:10.3389/fcvm.2025.1568844)
Supplement: Supplementary file 3 [file Datasheet3.docx]

Did not answer 14-day follow-up call (N=5)

Did not meet eligibility criteria (N=49)

Did not answer clinical follow-up call (N=51)

Chose not to participate in survey evaluation (N=48)

- Inexperienced with technology, or no computer (N=25)
- Exercise on their own (N=17)
- Feel unwell (N=4)
- Language barrier (N=2)

Answered 14-day follow-up call and completed the survey (N=112)

Agreed to complete survey (N=117)

Answered clinical follow-up call (N=165)

Eligible for JUMPSTART program (N=216)

TAVR patients in overnight model during study period (N=265)

**Supplemental File 3**. JUMPSTART pilot evaluation recruitment and participation.
